# A Multifunctional Core–Shell Nanoemulsion-Mediated Disruption of Asphaltene Aggregates for Unconventional Reservoir Oil Recovery Enhancement

**DOI:** 10.3390/molecules31091475

**Published:** 2026-04-29

**Authors:** Meng Cai, Qingguo Wang, Lichao Wang, Zhixuan Zhu, Jianxun Meng, Yanqiu Fang, Shangfei Wang, Lihong Yao, Qi Lv, Qi Zhou, Wenjing Li

**Affiliations:** 1 Daqing Oilfield Production Technology Institute, Daqing 163453, China; 2 Northeast Petroleum University, Daqing 163318, China; 3 Heilongjiang Provincial Key Laboratory of Oil and Gas Reservoir Stimulation, Daqing 163453, China; 4 No. 1 Production Plant, Daqing Oilfield Limited Company, Daqing 163001, China

**Keywords:** oil displacement agent, nano-emulsion, asphaltene, molecular dynamics, enhanced oil recovery

## Abstract

The development of tight heavy-oil reservoirs is severely hampered by the high viscosity and poor mobility of crude oil caused by strong intermolecular stacking interactions among asphaltenes, coupled with the substantial adsorption loss and inadequate deep transport capacity of conventional displacement agents. By targeted penetrant delivery, a novel nanoemulsion system with a well-defined “core–shell” architecture was synthesized to address these critical challenges. The physicochemical properties, stability and oil displacement performance were evaluated. The prepared nanoemulsion exhibited an ultrasmall and uniform particle size distribution between 10 nm and 20 nm. It also demonstrated exceptional dispersibility in aqueous media and remarkable thermal and salinity stability under reservoir conditions. Furthermore, an ultralow critical micelle concentration of approximately 0.01% could be achieved and the oil–water interfacial tension was reduced to 7.3 × 10^−2^ mN/m, significantly outperforming the conventional surfactant AES. Core flooding tests revealed that the proposed nanoemulsion enhanced oil recovery by 37.1% and attained a displacement efficiency of 68.9% in oil-wet capillary models. Molecular dynamics simulations further elucidated the underlying synergistic mechanism. The hydrophilic shell minimized adsorption on rock surfaces, facilitating deep migration within nanoporous channels. The hydrophobic core, containing terpinene as a penetrant, effectively disrupted the π-π stacking of asphaltenes due to its nonplanar molecular configuration. This disruption transformed the asphaltene aggregates from a tightly packed state to a dispersed state, resulting in substantial viscosity reduction. This work elucidated the mechanism of asphaltene aggregate disruption by nanoemulsions at the molecular level, offering a promising and theoretically grounded strategy for the efficient exploitation of tight heavy-oil reservoirs.

## 1. Introduction

Unconventional oil and gas resources are playing an increasingly important role in the global energy supply structure [1,2,3]. However, the economic and efficient development of such reservoirs faces inherent challenges. The complex microscopic pore structures severely limit fluid percolation capacity, while heavy components in crude oil, such as asphaltenes, lead to high viscosity and poor mobility [4,5]. Several critical challenges hinder the efficient development of tight heavy-oil reservoirs. Chief among them is the substantial adsorption loss of conventional surfactants during migration, which leads to rapid depletion of active components. This issue is further compounded by the poor stability of conventional nanomaterials under high-salinity and high-temperature conditions [6]. Furthermore, existing technologies lack the capability to chemically disrupt asphaltene aggregates, leaving the root cause of high viscosity unaddressed. Notably, the behavior of asphaltenes in nanoporous reservoirs extends beyond viscosity concerns to include thermodynamic phase stability. Capillary pressure effects in shale nanopores have been shown to substantially modify three-phase vapor–liquid–solid equilibria, causing asphaltene precipitation to occur at lower pressures and gas injection concentrations during CO_2_ flooding and CCUS operations [7]. Therefore, constructing a displacement system that combines deep transport, low adsorption loss, and efficient viscosity reduction is not only an urgent engineering need for the efficient extraction of unconventional reservoirs but also an expansion of the understanding of microscopic-scale multi-phase and multi-component interaction mechanisms, holding significant scientific and application value. This multifaceted nature of asphaltene-related challenges further justifies the development of multifunctional displacement agents capable of both deep transport and chemical remediation of asphaltene aggregates.

Extensive exploration has been conducted worldwide to address these challenges. Research has largely focused on the interfacial modification effects of single materials including inorganic nanoparticles, gradually progressing to composite systems with both interfacial and chemical regulation functions [8,9,10]. Nanoemulsion technology is regarded as a highly promising solution due to its unique nanoscale effects, excellent interfacial activity, and favorable transport capability in porous media. Inorganic nanoparticles such as SiO_2_ and Al_2_O_3_ can adsorb at the oil–water interface to stabilize emulsions and adjust the mobility ratio. They also promote the wettability alteration of rock surfaces from oil-wet to water-wet, thereby enhancing the oil washing and stripping efficiency [11,12,13,14]. Surfactants, such as the gemini and bola types, can achieve ultralow interfacial tension on the order of 10^−3^ mN/m, significantly improving the displacement process at the microscale. In addition, the high-temperature and high-salinity environment of reservoirs poses more stringent requirements on the stability, salt tolerance, and compatibility of the oil displacement agent systems. Meeting these demands has motivated extensive research into the design of stabilization mechanisms and functional architectures for performance optimization. A temperature-sensitive silica nanoparticle-stabilized Pickering emulsion has been developed [15]. This system can trigger a transition at a specific formation temperature of 80 °C, and the released particles can achieve efficient selective plugging in low-permeability and high-permeability cores, demonstrating its potential for fluid diversion applications. The introduction of Al_2_O_3_ nanoparticles can synergistically enhance the mechanical strength of the surfactant interfacial film and significantly improve the final oil recovery in core flooding experiments [16]. In terms of surfactant system design and innovation, the significant advantages of nanoemulsions over traditional micellar systems in spontaneous imbibition have been revealed through comparative studies, as their nanoscale droplets (70–100 nm) endow the system with lower interfacial tension and better pore-throat accessibility [17]. A nanoemulsion system with pH-responsive properties using biosurfactants has been constructed to provide a novel approach for controllable oil displacement and demulsification [18]. In addition to pH-responsive systems, other stimulus-responsive materials have also been applied for enhancing oil recovery. pH-responsive silicon quantum dots enable reversible emulsification and demulsification, achieving 14.65% enhanced oil recovery through alkali synergy [19]. Temperature-responsive Janus nanosheets with salt-tolerant properties achieve rapid emulsification at 65 °C and demulsification at 25 °C, improving heavy oil recovery by 13.2% [20]. CO_2_-switchable Pickering emulsions stabilized by Janus nanoparticles undergo fast phase separation upon CO_2_ injection [21]. Although these stimulus-responsive systems offer controllable emulsification and demulsification, their primary functions remain interfacial tension reduction and wettability alteration, with the responsive behavior mainly serving operational convenience rather than chemically addressing asphaltene aggregates. Surface-active ionic liquids can serve as efficient emulsifiers, with the prepared nanoemulsions exhibiting excellent long-term stability and wettability alteration capability under high-temperature and high-salinity environments [22]. In terms of mechanism exploration and theoretical modeling, the pioneering work in compositional model construction and history matching has laid a theoretical foundation for quantitatively describing and predicting the complex flow behavior of nanoemulsions in porous media [23]. Experimental studies have elucidated that polymer–surfactant–nanoparticle ternary composite systems can enhance the rheological properties and oil miscibility by forming unique supramolecular structures, thereby synergistically improving macroscopic sweep efficiency and microscopic oil-washing efficiency [24]. Notably, a groundbreaking concept of preparing low-concentration nanoemulsions using crude oil itself as the dispersed phase has been proposed, achieving significant results in microscopic displacement experiments, pointing towards a new direction for developing low-cost, high-efficiency systems [25]. In parallel with these developments in displacement agent formulation, considerable progress has also been made in understanding the fundamental behavior of asphaltenes in nanoporous media. Recent experimental work has quantified asphaltene deposition during CO_2_ flooding, with NMR T_1_–T_2_ spectroscopy revealing that miscible CO_2_ injection can induce deposition percentages as high as 50.64% and permeability reductions of up to 65.25% in low-permeability sandstones [26]. Molecular dynamics simulations have further elucidated the structural drivers of asphaltene aggregation, showing that extended aromatic cores and heteroatom-containing aliphatic chains promote strong aggregation, and that molecular polydispersity can exert both synergistic and antagonistic effects [27]. Thermodynamic models incorporating capillary pressure have demonstrated that nano-confinement significantly alters three-phase vapor–liquid–solid equilibria, shifting asphaltene precipitation to lower pressures during CO_2_ injection [28]. Complementary machine learning approaches have enabled accurate prediction of precipitation amounts across diverse crude oil samples [29,30], while recent reviews have synthesized these findings, emphasizing the time-dependent kinetics of precipitation and the promise of nanomaterials for aggregation inhibition [31,32]. Collectively, these studies underscore the multifaceted nature of asphaltene-related challenges in unconventional reservoirs. Nevertheless, within nanoemulsion design, the capability to chemically disrupt pre-existing asphaltene aggregates in the reservoir remains largely unaddressed, whereas asphaltene research has mostly focused on singular functions like precipitation inhibition. Despite these advances, most reported nanoemulsion systems have been designed to achieve a single function, such as interfacial tension reduction and wettability alteration. A synergistic design strategy that integrates deep migration capability, minimized interfacial adsorption, and chemical intervention on asphaltene aggregates within a single platform remains absent.

However, significant shortcomings remain when current nanoemulsion systems face the complex environments of unconventional reservoirs. Most existing systems are designed to achieve a single function. A synergistic design strategy that integrates deep migration capability, minimized interfacial adsorption, and efficient chemical intervention on heavy crude oil components such as asphaltenes remains absent. Although progress has been made in composite systems combining nanomaterials and surfactants, as well as emulsification and viscosity-reduction systems, physical blending often encounters issues such as insufficient compatibility, weak functional synergy, and poor long-term stability. A fundamental understanding of the molecular-scale interactions among nanoparticles, surfactants, and heavy crude oil components such as asphaltenes is still lacking. Hence, there is an urgent need for synergistic studies that combine molecular dynamics simulations with systematic experimental validation.

In this paper, a novel multifunctional nanoemulsion with a core–shell structure was designed and prepared. The particle size distribution, dispersibility, thermal and salt stability of the system were systematically characterized, followed by an evaluation of its interfacial regulation capability and oil displacement efficiency through core flooding experiments. Combining molecular dynamics simulations with experimental validation, the mechanisms underlying its reduced adsorption, enhanced deep migration, and asphaltene deaggregation were elucidated. The findings establish a multifunctional nanoemulsion platform that integrates deep transport, interfacial modification, and rheological control, providing a promising and mechanistically grounded strategy for the efficient development of unconventional reservoirs.

## 2. Results and Discussion

### 2.1. Structure and Basic Properties of the Nanoemulsion

**Figure 1:**
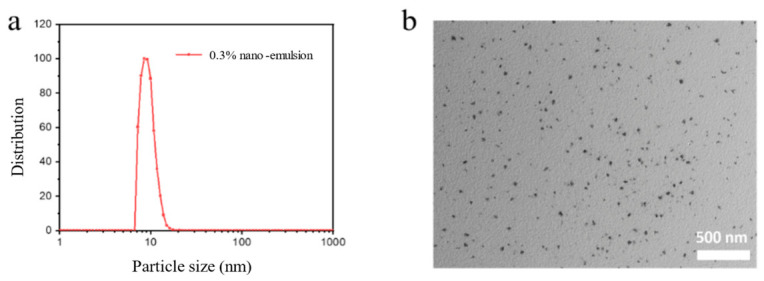
Hydrated particle size and micro-structure of the nanoemulsion. (**a**) DLS results for particle size (**b**) TEM picture.

The dynamic light scattering (DLS) test results were shown in Figure 1a. The hydrated particle size of the nanoemulsion was mainly distributed between 10 and 20 nm, with a peak around 15 nm, showing a single, narrow peak, indicating uniform particle size and good dispersibility within the system. TEM images (Figure 1b) further verified the morphological characteristics of the nanoparticles. The particles exhibit a near-spherical structure overall, with sizes generally less than 20 nm, consistent with the DLS results. The agreement between DLS and TEM results confirmed that the prepared nanoemulsion indeed possessed typical nanoscale features with uniform and stable structure. This ultra-small particle size characteristic provided the foundation for its effective penetration into micro–nano-sized pore-throats and the enhancement of deep sweep volume in unconventional reservoirs.

**Figure 2:**
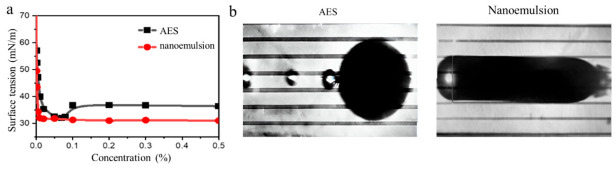
Comparison of interface properties between nanoemulsion and conventional surfactant AES (**a**) surface tension and (**b**) interfacial tension.

Interfacial performance was an important indicator for evaluating the efficiency of an oil displacement agent system. As shown in Figure 2a, the critical micelle concentration (CMC) of the prepared nanoemulsion system was approximately 0.01%, significantly lower than that of the conventional surfactant AES (0.1%). The lower CMC indicated that this system could form stable micellar structures and exhibited high interfacial activity at lower concentrations. This phenomenon was mainly attributed to the synergistic effect between the organic penetrant and the surfactant molecules, making the hydrophobic segments more prone to aggregation. When the concentration exceeded the CMC, the nanoemulsion molecules could form an ordered adsorption film at the oil–water interface, effectively reducing interfacial energy and displacement resistance. As shown in Figure 2b, the nanoemulsion achieved an interfacial tension of 7.3 × 10^−2^ mN/m with crude oil at 70 °C. This value is significantly lower than that of the AES system and well below the typical threshold of 2 mN/m required for conventional oil recovery chemicals. This result indicated that the nanoemulsion system could significantly weaken the capillary force at the oil–water interface, reduce the interfacial confinement energy of residual oil, and promote the detachment and migration of trapped crude oil, thereby providing an interfacial chemical basis for improving oil displacement in unconventional reservoirs.

**Figure 3:**
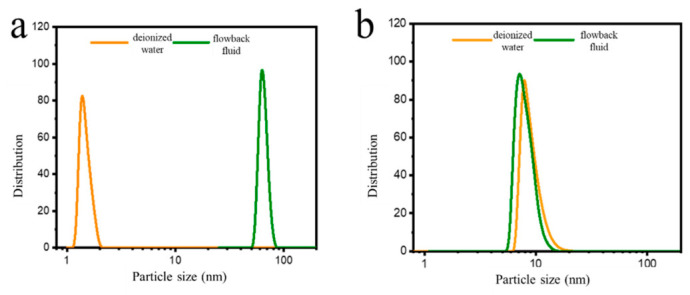
Size changes in oil displacement agents in deionized water and flowback fluid. (**a**) AES; (**b**) nanoemulsion.

As shown in Figure 3, the micelle size of the nanoemulsion and AES systems in aqueous solution and their stability under reservoir conditions were compared. The characteristic particle size of the AES system was less than 3 nm, indicating that it primarily exists in a dynamic equilibrium between small-scale micelles and single-molecule dissolved states in the aqueous phase. The AES system exhibited stronger overall hydrophilicity due to the longer and bulkier hydrophilic groups of its molecules. As a result, the self-assembly driving force was insufficient to maintain large stable aggregates, making it difficult to form micelles with larger size and stable morphology. In contrast, the nanoemulsion containing the organic penetrant induced the surfactant to form stable aggregates with a size of about 10 nm, demonstrating higher structural stability in both scale and morphology. In high-salinity flowback water, inorganic salts significantly affected the hydrophilic–lipophilic balance of AES, readily promoting the formation of secondary aggregates larger than 50 nm. Such aggregates posed a risk of pore-throat plugging or deactivation of the surface-active components. Relying on its “core–shell” structure, the nanoemulsion system could control the particle size within the range of about 10 nm in both deionized water and flowback water, exhibiting excellent salt tolerance. The surface tension and interfacial tension of the samples exhibited no significant variation after heating (Table 1), indicating that the nanoemulsion agent maintained good stability under thermal conditions.

The results in Table 2 revealed that the cuttings dispersion rate of the clear water system was 7.35%, indicating that the selected core exhibited strong water sensitivity. With the introduction of 0.1% nanoemulsion agent, the cuttings dispersion rate was reduced to 4.33%, thereby meeting the specified requirement. These findings indicated favorable compatibility between the nanoemulsion system and the reservoir rock. The reduction in dispersion rate was primarily related to the interfacial regulation capability and system stability of the nanoemulsion system. On the one hand, by adjusting the rock surface wettability to a neutral or weakly hydrophilic state, the surface-active components in the nanoemulsion reduced water invasion into clay mineral layers and mitigated their stripping, inhibiting cuttings disintegration and separation. On the other hand, the nanoemulsion exhibited high salt stability, maintaining a stable micellar structure under high-salinity conditions. This stability prevented inorganic salts from disrupting the surface electrical balance, thus preserving the charge layer on the rock surface and suppressing particle separation induced by electric double-layer compression. Furthermore, the low adsorption characteristic of the system ensured that the active components could continuously exert wettability modification and anti-sensitivity effects within the pore-throats. Overall, the nanoemulsion agent effectively mitigated rock water sensitivity without increasing adsorption loss, thereby enhancing reservoir compatibility and long-term stability.

### 2.2. Evaluation of Wettability Regulation and Oil Displacement Performance

**Figure 4:**
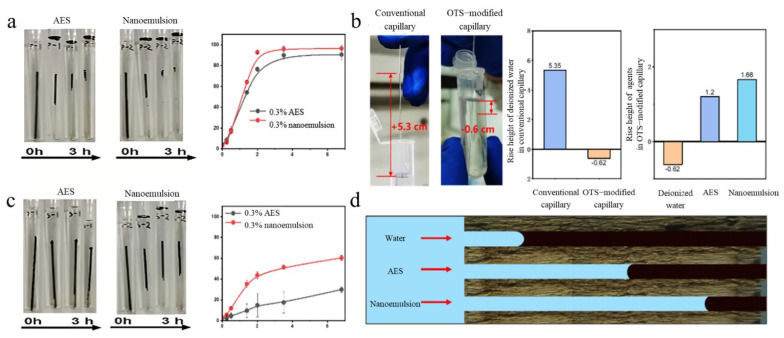
Wetting properties and displacement effects of AES and nanoemulsions: (**a**) oil displacement rate of AES and nanoemulsions in the hydrophilic capillary; (**b**) percolation height of displacement agent in OTS modified hydrophobic capillary; (**c**) oil displacement rate of AES and nanoemulsions in the hydrophobic capillary; (**d**) schematic diagram of swept crude oil in channels by water, AES, and nanoemulsions.

Figure 4a revealed that the AES system demonstrated considerable displacement efficiency in the hydrophilic capillary model. An OTS-modified capillary model was constructed to more closely approximate the wettability environment of oil-wet micro-channels in reservoirs, as shown in Figure 4b. The average rise height of deionized water in the unmodified capillary was 5.35 cm, indicating a hydrophilic inner surface. The liquid level dropped after OTS treatment and the rise height was −0.62 cm, indicating that the capillary exhibited hydrophobic and oil-wet characteristics towards the aqueous phase. In the oil-wet model shown in Figure 4c, after 7 h of displacement, the oil displacement efficiency of AES was 31.1%, while that of the nanoemulsion reached 68.9%. Figure 4d further indicated that within hydrophobic and oil-wet channels, the aqueous phase struggled to wet the inner wall and generate effective capillary driving force, making it difficult for ordinary water flooding to enter the pore-throats. In contrast, both AES and the nanoemulsion could improve the interfacial wettability state in oil-wet channels, achieving penetration and displacement of trapped oil, with the nanoemulsion demonstrating higher penetration capability. Integrating the capillary rise and displacement results, it could be concluded that the nanoemulsion reduced the capillary pressure barrier in oil-wet channels by lowering interfacial tension and regulating wettability, thereby enhancing both sweep volume and percolation depth, ultimately achieving higher oil displacement efficiency than AES within the same displacement time (Figure 4d). The above results provide experimental support for the applicability of the nanoemulsion in unconventional reservoirs dominated by oil-wet and micro-pore-throat characteristics.

**Figure 5:**
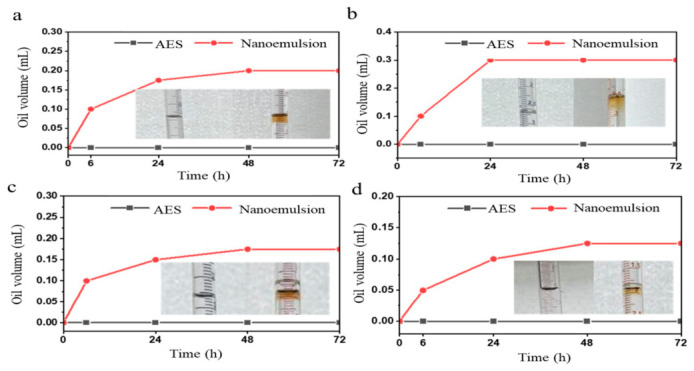
Comparison of spontaneous permeability draining recovery rate of core samples. (**a**) *k* = 0.11 mD (**b**) *k* = 0.19 mD (**c**) *k* = 0.30 mD (**d**) *k* = 0.41 mD.

As shown in Figure 5, in spontaneous imbibition experiments using oil-wet cores, the 0.3% nanoemulsion system demonstrated significantly superior oil displacement performance compared to the AES system at the same concentration. The ultimate recovery factor was markedly higher, with calculation results showing that the nanoemulsion system increased the oil recovery by approximately 37.1% compared to AES. This result indicated that the nanoemulsion could more effectively overcome capillary trapping and wettability reversal effects in oil-wet porous media, achieving further mobilization of residual oil. This enhancement was primarily attributed to the composite driving effects of the nanoemulsion system. The relatively low interfacial tension effectively weakened the capillary trapping force at the oil–water interface. Simultaneously, the nanoemulsion possessed excellent dispersibility and low adsorption characteristics, enabling it to penetrate deep into pore-throats and continuously act on residual oil interfaces.

### 2.3. Multi-Scale Elucidation of the Stimulation Mechanism of Nanoemulsion from Macroscopic Performance to Molecular Behavior

**Figure 6:**
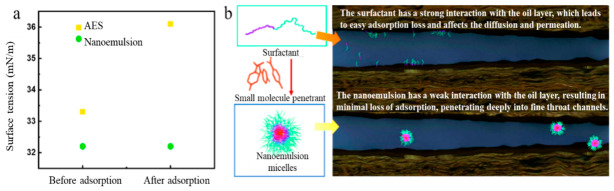
Adsorption behavior of AES and nanoemulsion (**a**) surface tension before and after adsorption; (**b**) schematic diagram of adsorption loss in the oil layer.

Figure 6a demonstrated that both the surface tension and interfacial tension of the AES solution increased significantly after interaction with rock pore-throats, indicating strong interfacial interactions of its active components on the rock surface, which led to attenuation of the interfacial activity of the AES system. In contrast, the surface tension of the nanoemulsion system remained almost unchanged, indicating that its interfacial properties could be stably maintained during contact. This difference reflected a fundamental distinction in the molecular interaction mechanisms of the two systems at the pore-throat interface. In the AES system, surfactant molecules were prone to adsorption under the influence of the rock surface, causing the effective concentration of active components to decrease rapidly with seepage depth. Capillary confinement in nanopores had been demonstrated to influence asphaltene precipitation behavior, while the nanoemulsion system developed herein targeted the converse mechanism of chemical disruption for already formed asphaltene aggregates. The nanoemulsion system, owing to its structural stability and interfacial compatibility, maintained high interfacial activity even after contact with the rock, thereby enabling deep transport of effective components.

The performance advantages of the core–shell nanoemulsion over conventional surfactant–solvent blends and previously reported Pickering emulsions were evident in both structural design and quantitative metrics. Unlike simple surfactant–solvent mixtures in which the solvent component remained unprotected and was susceptible to premature loss, the core–shell architecture encapsulated terpinene within a hydrophobic core shielded by a hydrophilic polyoxyethylene shell [33]. This configuration minimized interfacial adsorption and enabled targeted release of the penetrant upon contact with heavy oil in the deep reservoir. After contact with oil sands, the surface tension of the nanoemulsion remained nearly unchanged, indicating minimal adsorption loss of active components, whereas the AES system exhibited a marked increase under identical conditions. This difference was particularly significant given that surfactant retention on rock surfaces was a well-documented limitation in chemical EOR processes [34]. The nanoemulsion achieved an ultralow critical micelle concentration of approximately 0.01%, an order of magnitude lower than that of AES (~0.1%), and reduced the oil–water interfacial tension to 7.3 × 10^−2^ mN/m. In core flooding tests using oil-wet capillary models, the nanoemulsion enhanced oil recovery by 37.1% compared to the AES system. Furthermore, unlike Pickering emulsions stabilized by rigid inorganic nanoparticles that posed a risk of pore-throat plugging in nanoporous media, the present soft-matter nanoemulsion exhibited a uniform particle size distribution between 10 nm and 20 nm, ensuring superior pore-throat accessibility and deep migration capability. Moreover, whereas previously reported nanoemulsion systems had largely focused on interfacial tension reduction or wettability alteration, the present core–shell nanoemulsion uniquely integrated these interfacial functions with targeted chemical disruption of asphaltene aggregates.

Unconventional reservoirs generally exhibited significant oil-wet characteristics. When conventional surfactant molecules entered oil-bearing pore channels, their hydrophobic ends tended to interact with the oil film on the pore walls, leading to gradual accumulation and adsorption of molecules on the pore-throat surfaces. As seepage progressed, the adsorption loss of surfactant accumulated continuously. The effective concentration of the system in deep regions became insufficient to maintain low interfacial tension, resulting in increased capillary pressure, weakened displacement capability and difficulty in further mobilizing residual crude oil. In contrast, the seepage behavior of the nanoemulsion system was markedly different. Its low adsorption characteristic was closely related to the “core–shell” type micelle structure (Figure 6b). The core of the nano-micelle consisted of surfactant hydrophobic groups and the organic penetrant, while the outer layer was composed of hydrophilic groups, forming aggregates that were stable under reservoir conditions. This structure significantly weakened the interaction between the outer shell of the micelle and the oil-wet pore walls, thereby avoiding strong adsorption, while relying on the hydration layer and steric hindrance effects to maintain interfacial stability.

As the nano-micelles migrated deeper with the seepage flow, the content of heavy oil components in the pores gradually increased. The interaction between the hydrophobic penetrant in the core and the oil phase strengthened, causing relaxation of the local micelle structure and gradual release of the penetrant. The released organic penetrant could disrupt the π-π stacking structure between asphaltene molecules and weaken the intermolecular forces within aggregates. Consequently, the asphaltene stacking was locally disrupted and crude oil viscosity was reduced, thereby enhancing its mobility and transportability [35]. This process manifested as a synergistic mechanism of stable transport and targeted release. The nanoemulsion could maintain relatively low adsorption to achieve deep seepage and efficient displacement at the macroscopic scale, while the action of the organic penetrant within the nanoemulsion achieved rheological regulation of the oil phase at the molecular scale. The synergistic effect of these two aspects constituted the key mechanistic foundation for the nanoemulsion system to achieve efficient oil displacement and also provided multi-scale theoretical support for understanding its stimulation behavior under complex reservoir conditions.

**Figure 7:**
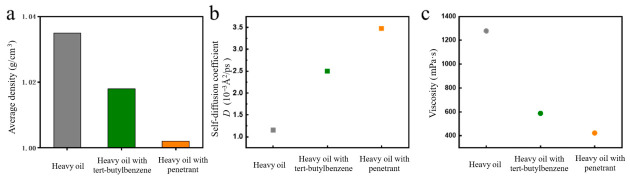
Properties of heavy oil with additions (**a**) average density (**b**) self-diffusion coefficient (**c**) viscosity.

Table 3 indicated that the penetrant molecules contained within the nanoemulsion agent exhibited excellent crude oil viscosity reduction performance, thereby significantly improving oil flowability. Taking the crude oil from the block with the permeability of 0.11 mD as an example, without adding any organic small molecules, the crude oil had high viscosity and almost no fluidity. After adding tert-butyl benzene or the penetrant, its fluidity was noticeably enhanced. Among them, the penetrant exhibited a better viscosity-reducing effect on the crude oil than tert-butyl benzene, with a relative increase in fluidity of about 30%. As shown in Figure 7a, the average density decreased after the introduction of tert-butyl benzene or the penetrant. Combined with the self-diffusion coefficient *D* in Figure 7b, the system’s fluidity was positively correlated with *D*. The results indicated that adding both tert-butyl benzene and the penetrant could effectively increase the self-diffusion coefficient of the heavy oil system, reduce the density and viscosity (Figure 7c), thereby enhancing the fluidity of the heavy oil. Compared to tert-butyl benzene, the penetrant exerted a more pronounced improvement on the fluidity of heavy oil. These results presented in Figure 7 and Table 3 confirmed that the penetrant itself possessed inherent viscosity-reducing capability under static mixing conditions. However, a penetrant alone would be rapidly depleted by adsorption onto rock surfaces before reaching deep reservoir zones under dynamic transport conditions within porous media, as evidenced by the surface tension increase observed for the unprotected AES system in Figure 6a. In contrast, the core–shell architecture protected the penetrant during transport through the hydrophilic shell, minimized interfacial adsorption, and enabled targeted release upon contact with heavy oil. The 37.1% enhanced oil recovery achieved by the nanoemulsion over AES therefore reflected the synergistic contribution of low adsorption transport, targeted penetrant release, and asphaltene aggregate disruption, rather than merely the presence of the organic solvent.

**Figure 8:**
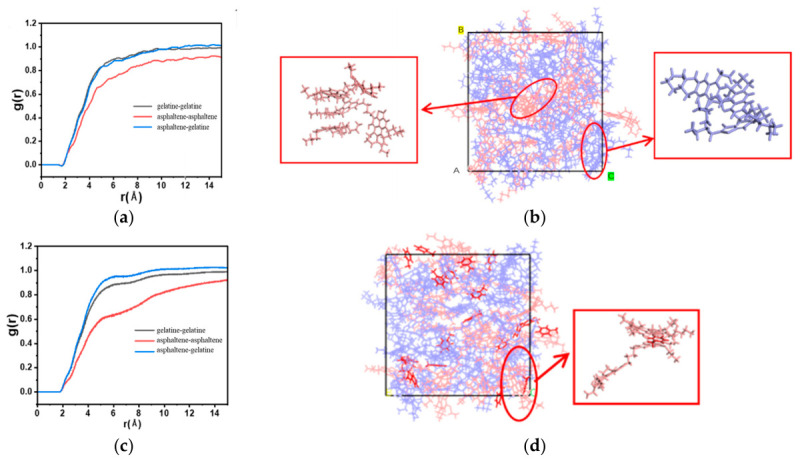

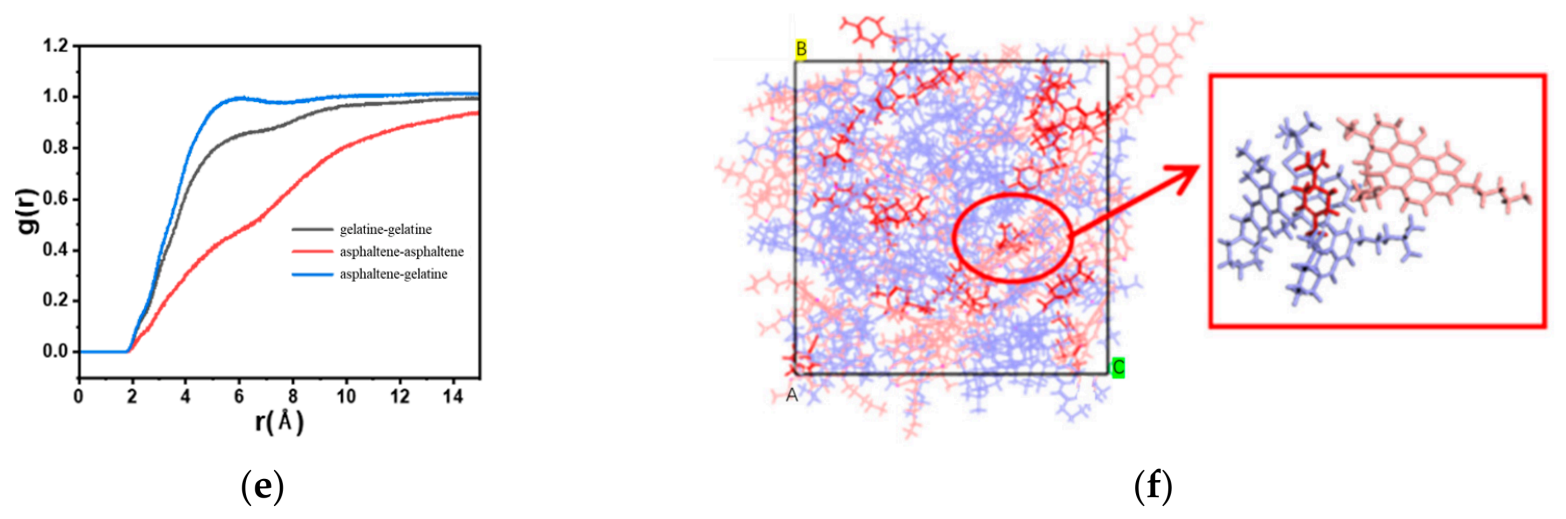
Radial distribution function of crude oil with additions: (**a**) radial distribution function of the crude oil system; (**b**) π-π stacking between asphaltenes and asphaltenes, and between kerogens and kerogens in the crude oil system; (**c**) radial distribution function of the crude oil with isopropylbenzene system; (**d**) schematic diagram of the interaction between tert-butylbenzene and the crude oil components; (**e**) radial distribution function of the crude oil with solvent system; (**f**) schematic diagram of the interaction between the solvent and the crude oil components.

The interactions among components within the three systems were characterized and analyzed using the radial distribution function *g*(r), with results shown in Figure 8. Point O represented the coordinate origin, and axes A, B, and C corresponded to the x, y, and z spatial dimensions. The yellow region was labeled B and the green region was labeled C in Figure 8. Figure 8a revealed that in the pure crude oil system, components formed tight aggregates through π-π stacking. This observation was consistent with molecular dynamics studies which had established that π-π stacking between the aromatic cores of asphaltene molecules constituted a primary driving force for nano-aggregation [36]. The strength of this stacking interaction was governed by the molecular architecture of the asphaltenes, particularly the size of the polyaromatic core and the presence of heteroatoms, with extended aromatic systems exhibiting significantly stronger aggregation tendencies. This phenomenon arose because asphaltene molecules had a nearly planar structure and high molecular weight, making them prone to stacking the π-π interactions, leading to increased viscosity and reduced fluidity of the crude oil system (Figure 8b). Figure 8c illustrated that after the addition of tert-butylbenzene, its molecules intercalated between asphaltene and gelatine molecules and established π-π interactions with them. Consequently, the stacking between asphaltene molecules was partially disrupted (Figure 8d), resulting in enhanced crude oil flowability. Tert-butyl benzene could only attenuate the intensity of this peak and was difficult to completely break the associative structure between crude oil components. In contrast, as shown in Figure 8e, after adding the penetrant, this characteristic peak almost completely disappeared, indicating that the penetrant could effectively disrupt the stacking structure of asphaltene molecules. Penetrant molecules could deeply penetrate between asphaltene and gelatine molecules, significantly weakening the stacking and associative interactions among heavy oil components. Its disruption effect might be related to the non-planar three-dimensional configuration of the molecules. Penetrant molecules possessed a steric effect, and their molecular motion had higher disorder and rotational freedom. These penetrant molecules disturbed the ordered stacking structure of asphaltenes, promoted a transition of the crude oil system from a tightly aggregated state toward a more dispersed state, and significantly enhanced crude oil fluidity (Figure 8f). The observed disruption of π-π stacking by the penetrant was consistent with recent studies demonstrating that certain molecular additives could weaken aromatic stacking interactions. RDF analysis indicated that sophorolipids significantly weakened π-π stacking among asphaltene molecules, reducing total interaction energy and aggregate size [37]. Similarly, certain polar functional groups could disrupt π-π stacking and hinder asphaltene aggregation [38].

**Figure 9:**
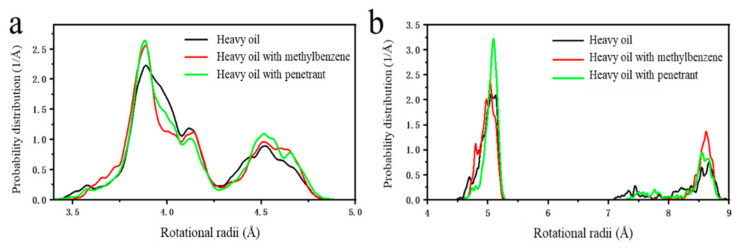
Changes in the probability distribution of the rotational radii before and after adding additions of (**a**) gelatine components and (**b**) asphaltenes components.

The distribution of radius of gyration for asphaltene and gelatine components in the systems was calculated to further reveal the changes in the degree of entanglement between asphaltene and gelatine molecules after adding the penetrant, with results shown in Figure 9. Due to the smaller molecular weight of gelatine molecules, the change in their radius of gyration was not significant. However, the radius of gyration of asphaltene molecules exhibited an increasing trend after adding both tert-butylbenzene and the penetrant, indicating a shift in asphaltene molecular conformation from tightly entangled toward a more extended arrangement, effectively alleviating intermolecular entanglement [39]. The penetrant molecules in the nanoemulsion system could penetrate between asphaltenes and gelatines, weakening the stacking and entanglement between them, allowing crude oil components to be stripped and re-dispersed, thereby significantly improving crude oil fluidity. This disruption and disentanglement process indicated that the viscosity reduction effect of the nanoemulsion system did not originate from simple dilution but rather from significantly altering the microscopic aggregation structure of crude oil components to achieve rheological property improvement.

**Figure 10:**
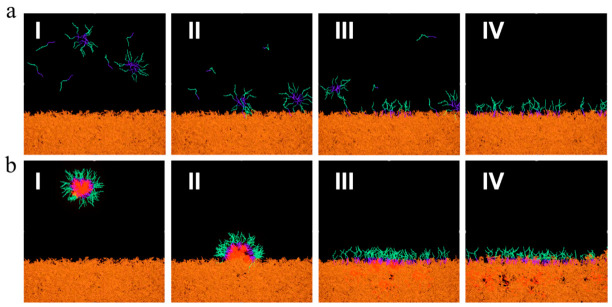
Schematic diagram of the interaction between oil displacement agents and reservoir crude oil: (**a**) surfactant (I) injection; (II) contact between surfactant and oil layer; (III) disassembly; (IV) adsorption; (**b**) nanoemulsion enhancer (I) injection; (II) contact between nanoemulsion and oil layer; (III) release of de-associating small molecules by nanoemulsion; (IV) displacement.

Figure 10 depicted a schematic diagram of the working process of the nanoemulsion in unconventional reservoirs. Firstly, the hydrophilic shell ensured the long-distance transportation and low adsorption of the nano-micelles in oil-wet channels. Moreover, the ultra-low interfacial tension effectively overcame capillary forces, reducing the confinement energy of residual oil. When the developed nanoemulsion system reached deep zones containing rich crude oil resources, the hydrophobic penetrant in the core was released. Through the three-dimensional configuration, it unlocked the entangled structure of asphaltene molecules, fundamentally reducing viscosity and enhancing crude oil fluidity. The synergistic action of these three steps ultimately achieved efficient mobilization of crude oil in unconventional reservoirs, enabling deep and efficient extraction [40,41].

As shown in Figure 10a, when conventional surfactants encountered crude oil in the oil layer, their hydrophobic ends interacted with the oil layer, leading to adsorption of surfactant molecules on the oil layer surface, lacking further interaction with the crude oil. The long hydrophilic chains of surfactants also made it difficult for them to further penetrate the oil layer to interact with crude oil components, thus making it challenging to strip crude oil from the rock formation. This limitation was directly evidenced by the surface tension increase observed for the AES system after contact with rock surfaces in Figure 6a, which indicated strong adsorption and depletion of active components during transport. In the nanoemulsion stimulant, the surfactant and organic small-molecule penetrant were dispersed in water as stable nano-micelles. Figure 10b illustrated that the organic penetrant and surfactant of the nanoemulsion formed stable “core–shell” type nano-micelles. As the nano-micelles flowed through oil-wet pore channels, their highly hydrophilic outer layer reduced adsorption loss on the reservoir rock, allowing the nanoemulsion to be transported into the channels of deeper oil layers, as confirmed by the near-constant surface tension of the nanoemulsion after rock contact in Figure 6a. When encountering deep crude oil, the oleophilic nature of the organic penetrant molecules and the hydrophobic ends forming the nanoemulsion began to interact more preferentially with the crude oil. Upon encountering deep crude oil, the oil affinity of the organic penetrant molecules and the hydrophobic ends forming the nanoemulsion began to drive interactions with the crude oil. The nano-micelles subsequently released the internal organic penetrant molecules, which permeated into the crude oil and interacted with asphaltene and gelatine components. This targeted release and the subsequent disruption of asphaltene aggregates were supported by the radial distribution function analysis in Figure 8, which demonstrated the near-complete disappearance of the characteristic π-π stacking peak after penetrant addition. The enhanced oil recovery of 37.1% achieved by the nanoemulsion over AES, as shown in Figure 5, further corroborated that this molecular-level disruption translated into macroscopic displacement efficiency. This process promoted the de-entanglement and disruption of asphaltenes, simultaneously reducing the viscosity and enhancing the fluidity of the crude oil, thereby promoting the stripping and migration of crude oil from the rock formation and enhancing oil recovery.

## 3. Materials and Methods

### 3.1. Materials and Instruments

Formation water with the salinity of 13,798 mg/L and the hardness of 7740.7 mg/L were used to prepare solutions. The crude oil used in this study was collected from a block in an unconventional reservoir, with a density of 0.82 g/mL. Four core samples with permeabilities of 0.11 mD, 0.19 mD, 0.30 mD, and 0.41 mD were used for physical simulation experiments. Oil sands were prepared by crushing these core samples. Sodium lauryl ether sulfate (AES, 70% purity) purchased from Shandong Yousuo Chemical Technology Co., Ltd., Linyi, China, octadecyltrichlorosilane (OTS, 90% purity) purchased from Shanghai Aladdin Biochemical Technology Co., Ltd., Shanghai, China. terpinene with the purity of 95% obtained from Anhui Energy Chemical Co., Ltd., Hefei, China, and alkyl polyoxyethylene surfactant (98% purity) supplied by Shandong Jining Sanshi Biotechnology Co., Ltd., Jining, China were used to prepare the nanoemulsion displacement agent in this work.

The instruments used to characterize the properties of the nanoemulsion system are shown as follows. Dynamic light scattering analyzer, NanoBrook 90Plus PALS (Brookhaven Instruments, Holtsville, NY, USA); Transmission electron microscope (TEM), Hitachi H-7650 (Hitachi High-Technologies Corporation, Tokyo, Japan); Surface tensiometer, Tensiometer K9 KRÜSS GmbH (KRÜSS GmbH, Hamburg, Germany); Spinning drop ultra-low interfacial tensiometer, KRÜSS TX500C (USA KINO Scientific Instrument Inc., Boston, MA, USA).

### 3.2. Construction and Characterization of the Novel Multifunctional Nanoemulsion System

Exactly 4.0 g of terpinene was thoroughly mixed with 16.0 g of alkyl polyoxyethylene surfactant using a magnetic stirrer (IKA C-MAG HS7 digital, IKA-Werke GmbH & Co. KG, Staufen im Breisgau, Germany) operating at a constant speed of 1000 rpm at 25 °C to form a homogeneous pre-mixed phase. This pre-mixed solution was subsequently introduced into 46.5 g of deionized water at a controlled addition rate under continuous stirring at the same speed. Stirring was maintained for an additional 0.5 to 1.0 h until the system spontaneously transformed into a homogeneous, transparent, and kinetically stable nanoemulsion. The resulting product was collected as the target nano-displacement agent.

Then the particle size and morphology of the prepared nanoemulsion system were characterized. The average particle size and distribution of the nanoemulsion were determined by dynamic light scattering (DLS) using deionized water as the dispersion medium. Before testing, samples were diluted to an appropriate concentration with deionized water and left to stand for 10 min to eliminate the effect of bubbles. Samples were dropped onto carbon-coated copper grids and allowed to dry naturally, then the microstructure was observed using transmission electron microscopy (HITACHI H-7650, Hitachi High-Technologies Corporation, Tokyo, Japan).

Nanoemulsion solutions with different mass fractions were prepared for determining the surface and interfacial properties. The equilibrium surface tension was measured using a surface tensiometer at 25 °C. Solutions of alkyl ether sulfate (AES) were tested under the same conditions for comparison.

The interfacial tension between the nanoemulsion and crude oil was measured using a spinning drop interfacial tensiometer. The test temperature was 70 °C. Crude oil was injected into the test solution as the dispersed phase. Centrifugal force generated by the rotation of the tube around the axis counteracted the effect of gravity on the droplet shape. After the oil droplet stabilized, images were captured and the interfacial tension was calculated by the accompanying CAST 4.0 software (USA KINO Scientific Instrument Inc.) using the spinning drop method, with the following equation applied.

(1) $$IFT=A\times P\times D^{3}\times \theta^{2}$$

where *IFT* was the interfacial tension, mN/m; *A* was a constant with the value of 0.000000144; *P* was the density difference between kerosene and the test solution, g/mL; *D* was the oil droplet diameter, mm; *θ* was the rotation speed, rpm.

### 3.3. Compatibility and Stability Evaluation of the Nanoemulsion

The nanoemulsion samples were placed at 70 °C for 6 h to evaluate the thermal stability of the system, after which the changes in surface tension and interfacial tension of the nanoemulsion prepared with formation water were measured.

The compatibility of the nanoemulsion stimulant with reservoir rock was evaluated using a cuttings dispersion rate test. A higher dispersion rate indicated more significant structural damage to the cuttings and poorer system compatibility. The core used in the experiment was crushed and sieved through a standard sieve. Cuttings with a particle size of 0.9–1.6 mm (12–20 mesh) were collected and dried at 105 °C. A total of 5.0 g of cuttings were weighed, and the initial mass *G*_0_ was recorded. The cuttings were placed into 50 mL of test solution and soaked at 70 °C for 4 h. After soaking, the cuttings were removed, rinsed with distilled water, filtered and dried. The sample was then sieved through a 0.56 mm (32 mesh) sieve to remove particles smaller than 32 mesh, and the mass *G*_1_ of the remaining cuttings after sieving was recorded. The cuttings dispersion rate was calculated using the following equation.

(2) $$D=\frac{G_{0}-G_{1}}{G_{0}}\times 100\%$$

where *D* was the dispersion rate.

Simultaneously, DLS was used to measure the micelle size of AES and the nanoemulsion in deionized water and formation water to characterize their stability.

### 3.4. Performance Evaluation of the Nanoemulsion System

An oscillating equilibrium method was used to determine the adsorption behavior of the nanoemulsion system on rock surfaces. A 100 mL conical flask was used for the experiment. The displacement medium and oil sand were added to the flask at a solid–liquid mass ratio of 1:15. After sealing, the flask was placed on a constant temperature shaker and oscillated at a constant speed for 24 h. After oscillation, the conical flask was removed, the supernatant was decanted, and then centrifuged at 3000 rpm for 3 min. The clear supernatant was taken to measure the change in surface tension. By comparing the difference in surface tension before and after the experiment, the adsorption loss of the nanoemulsion on the rock surface was characterized.

Then an Amott cell imbibition apparatus was used to evaluate the oil displacement performance of the nanoemulsion system. The cleaned core samples were dried overnight in a 70 °C oven. After taking out dried core samples the cooling them to the room temperature, the original weigh *m*_0_ was recorded. Subsequently, crude oil was injected under vacuum and the cores were soaked for 20 days to achieve full oil saturation. The saturated core was removed, and after wiping off surface residual crude oil, its mass was recorded as *m*_1_. The oil-saturated core was placed into an Amott cell with fine graduations. The displacing agents (0.3% conventional surfactant AES and the 0.3% nanoemulsion system) prepared using a 2% KCl solution as the base fluid were injected respectively, with the liquid level slightly above the lowest graduation. After sealing the cell, spontaneous imbibition experiments were conducted at a constant temperature of 70 °C. The volume *v* of oil spontaneously imbibed from the core was recorded at regular intervals until oil production ceased. The core oil production rate *f* was calculated using the following equation.

(3) $$f=\frac{\rho \cdot v}{m_{1}-m_{0}}\times 100\%$$

where *ρ* was the density of crude oil, g/mL; *v* was the volume of imbibed crude oil, mL.

**Figure 11:**
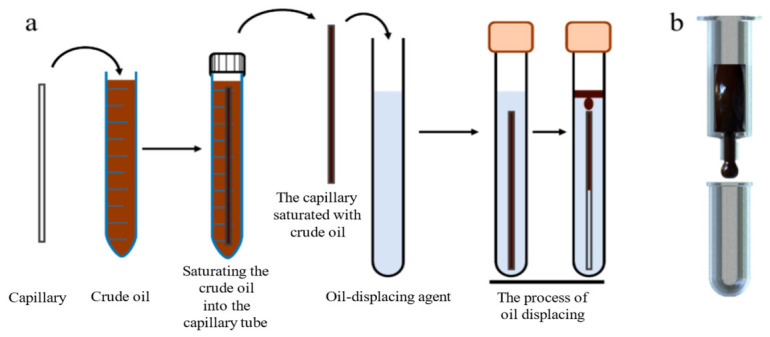
Evaluation schematic diagram of oil displacement performance; (**a**) schematic diagram of capillary oil displacement experiment; (**b**) schematic diagram of experimental setup for crude oil fluidity characterization.

OTS-modified quartz capillaries with the inner diameter of 0.5 mm and the length of 100 mm were used to simulate oil-wet channels, so that the capillary oil displacement performance of the nanoemulsion system could be characterized. Capillaries were cleaned with deionized water and dried as hydrophilic capillaries. Another batch of capillaries was soaked in a 5 × 10^−4^ g/mL OTS chloroform solution for 30 min, rinsed with trichloromethane, and dried to obtain OTS hydrophobic-modified capillaries. Both hydrophilic and hydrophobic capillaries were separately immersed in crude oil at 70 °C for 24 h to achieve full oil saturation. After taking out the two capillaries, surface crude oil was wiped off. The oil-saturated capillaries were then immersed into AES and nanoemulsion systems, respectively, for static oil displacement at 70 °C (as shown in Figure 11a). The change in oil column height within the capillary was observed and recorded at regular intervals. The oil displacement efficiency *f* was calculated based on the initial oil column height *h*_0_ and the remaining height *h*_t_ after displacement time *t*, using the following equation.

(4) $$R=\frac{h_{0}-h_{t}}{h_{0}}\times 100\%$$

As shown in Figure 11b, a 5 mL disposable syringe barrel with the needle removed was used as the outflow container to measure either the mass of crude oil flowing out within a specific time or the time required for complete outflow, thereby evaluating the improvement effect of the nanoemulsion system on crude oil fluidity. In the experiment, 3 g of crude oil was taken. Depending on the crude oil properties, 5% to 15% of organic small-molecule additive was added. After thorough mixing, the mixture was immediately transferred into the syringe barrel. The moment the first drop of oil began to fall was taken as the start time. For crude oils with lower fluidity, the mass of crude oil flowing out within 120 s was recorded. For crude oil with higher fluidity (Block L26), the time required for the complete outflow of the crude oil was used to characterize its fluidity. tert-Butylbenzene was set as the control group to compare the viscosity reduction and fluidity improvement effects of different systems. The mass of all added organic small molecules was the same.

### 3.5. Molecular Dynamics Simulation

**Figure 12:**
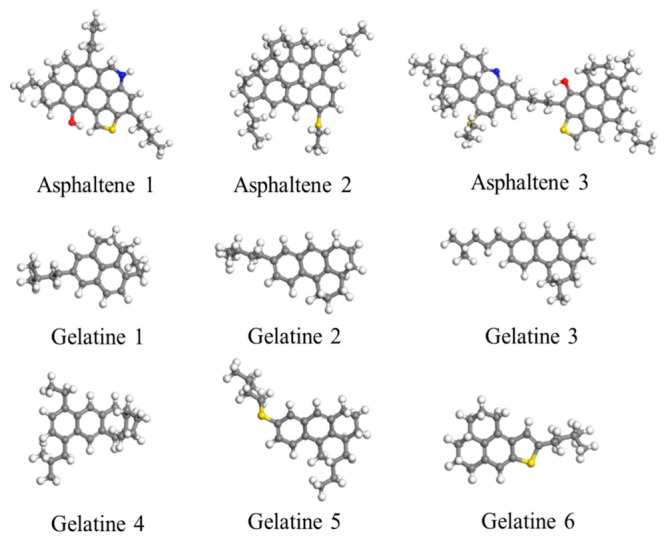
Molecular models of asphaltenes and gelatines.

Molecular dynamics simulations were conducted to reveal the adsorption loss mechanism of the targeted release nanoemulsion system, using Materials Studio software 2020. The disruption behavior of nanoemulsion towards asphaltenes in crude oil was performed to study the mechanism of action of the nanoemulsion on asphaltene and gelatine in the crude oil system. The Forcite module and the COMPASS II force field were selected to perform atomic-level molecular dynamics simulations. A simulation box with dimensions of 35 Å × 35 Å × 35 Å was constructed based on the Amorphous Cell module. The model contained asphaltene and gelatine molecules as shown in Figure 12 to form the crude oil system. According to the composition design in Table 4, tert-butylbenzene molecules and the nanoemulsion were introduced into the systems for comparison. After energy minimization, the system first underwent 500 ps of molecular dynamics simulation under the NPT ensemble, followed by 1000 ps under the NVT ensemble. The cutoff radius was set to 15.5 Å, the time step was 1 fs, and the pressure was 1 × 10^−4^ GPa (1 atm). Parameters such as the average density of the system, self-diffusion coefficient *D*, molecular radial distribution function *g*(r), and the radius of gyration distribution of molecules were calculated using the Forcite Analyze module to reveal the impact mechanism of the nanoemulsion on the aggregate structure and fluidity of crude oil molecules.

## 4. Conclusions

Aiming at key issues such as low permeability and insufficient displacement efficiency in the development of unconventional reservoirs, this study designed and constructed a novel nanoemulsion stimulation system with a “core–shell” structure. Systematic experiments and molecular simulation results indicated that the system had a particle size distribution concentrated between 10 nm and 20 nm, exhibited excellent dispersibility and salt stability, and possessed a critical micelle concentration as low as 0.01%. The interfacial tension as low as 7.3 × 10^−2^ mN/m of the developed nanoemulsion significantly outperformed that of conventional surfactant systems. The nanoemulsion could effectively reduce rock water sensitivity and adsorption loss, improve rock wettability, and achieve deep stable transport. When migrating in oil-wet pore-throats, it could significantly enhance oil recovery by maintaining low interfacial tension and wettability adjustment, with oil displacement efficiency increased by approximately 37.1% compared to the AES system. Molecular dynamics simulations further revealed that the hydrophobic penetrant within the emulsion core could disrupt the π-π stacking and entanglement structures between asphaltene molecules, promoting a transition of the crude oil system from a tightly aggregated state toward a more dispersed conformation, thereby reducing viscosity and enhancing fluidity. It was evident that this core–shell nanoemulsion system achieved deep mobilization and efficient recovery at the macroscopic scale through a three-step synergistic mechanism of low-adsorption transport, interfacial modification and molecular disentanglement. Moreover, the nanoemulsion system promoted a more dispersed molecular conformation of crude oil components, thereby enhancing fluidity at the molecular level. This study provides a new technical pathway and theoretical basis for the efficient development of unconventional reservoirs, demonstrating good potential for field application and promotion.

## Figures and Tables

**Table 1:** Thermal stability of nanoparticle emulsions.

Parameter	Before Heating	After Heating
Surface tension/mN/m	27.8	27.1
Interface tension/mN/m	0.1221	0.1348

**Table 2:** Core dispersal rate of nanoemulsion enhancing agent.

Sample	Number	*G*_0_/g	*G*_1_/g	*D*/%	Average *D*/%
Deionzed Water	1	5.0007	4.633	7.36	7.35
	2	5.0011	4.622	7.58	
	3	5.0001	4.6442	7.12	
0.1% Nanoemulsion	1	5.001	4.7715	4.59	4.33
	2	5.0009	4.8119	3.78	
	3	5.0004	4.7694	4.62	

**Table 3:** Viscosity reduction effect of penetrant.

Number	Test Item	No Addition	Tert-Butylbenzene	Penetrant	Addition Ratio (%)
1	Total time for complete flow/s	94	31	28	5
2	2 min flow rate of crude oil/g	0	1.4556	1.8883	10
3	2 min flow rate of oil/g	0	0.9323	1.2647	15

**Table 4:** Construction of molecular dynamics models for crude oil displacement.

System	Asphaltene Molecules	Gelatine Molecules	Other Molecules
Heavy oil	3 × 5	6 × 10	0
Tert-butylbenzene system used to displace heavy oil	3 × 5	6 × 10	Tert-butylbenzene 20
Nanoemulsion system used to displace heavy oil	3 × 5	6 × 10	Terpinene 20

## Data Availability

Data is contained within the article. The original contributions presented in this study are included in the article. Further inquiries can be directed to the corresponding author.
